# Dark chocolate intake and cardiovascular diseases: a Mendelian randomization study

**DOI:** 10.1038/s41598-023-50351-6

**Published:** 2024-01-10

**Authors:** Juntao Yang, Jiedong Zhou, Jie Yang, Haifei Lou, Bingjie Zhao, Jufang Chi, Weiliang Tang

**Affiliations:** 1 Department of Cardiology, Shaoxing People’s Hospital, Shaoxing, 312000 Zhejiang People’s Republic of China; 2 School of Medicine, Shaoxing University, Shaoxing, 312000 Zhejiang People’s Republic of China; 3 Department of Cardiology, Zhuji People’s Hospital, Zhuji, 311800 Zhejiang People’s Republic of China

**Keywords:** Hypertension, Nutrition

## Abstract

Previous intervention studies have shown some benefits of dark chocolate for the cardiovascular system, but it has not been established whether dark chocolate intake is associated with the risk of cardiovascular diseases (CVDs). To investigate the causality between dark chocolate intake and the risk of CVDs, a Mendelian randomization (MR) study was conducted. We obtained summary-level data on dark chocolate intake and CVDs from publicly available genome-wide association studies. In this MR study, the main approach was to use a fixed-effect model with inverse variance weighted (IVW) and evaluate the robustness of the results via sensitivity analysis. We found that dark chocolate intake was significantly associated with the reduction of the risk of essential hypertension (EH) (OR = 0.73; 95% CI 0.60–0.88; p = 1.06 × 10^−3^), as well as with the suggestive association to the reduced risk of venous thromboembolism (OR = 0.69; 95% CI 0.50–0.96; p = 2.81 × 10^−2^). However, no association was found between dark chocolate intake and the other ten CVDs. Our study provides evidence for a causality between dark chocolate intake and a reduced risk of EH, which has important implications for the prevention of EH in the population.

## Introduction

Cardiovascular diseases (CVDs) are the leading cause of death and a major contributor to disability in the global population, as well as a primary driver of the global disease burden. The mortality and prevalence of CVDs vary significantly across different regions of the world, with the highest prevalence in the Middle East and North Africa; the highest mortality in Central Asia and Eastern Europe, while relatively low in North America and Western Europe^1^. As of 2019, the total number of cases had reached 523 million (95% uncertainty interval: 497 million to 550 million)^1^. Thus, it is essential to identify both risk and protective factors for CVDs in daily life.

Chocolate is a globally popular food, and dark chocolate, due to its higher cocoa content, has a distinctive bittersweet flavor and contains more beneficial substances like flavanols and methylxanthines that are good for the body^2,3^. There are three main flavanols found in dark chocolate: procyanidin, catechin and epicatechin^2^. A number of mechanisms have been discovered that explain why these substances are beneficial for the cardiovascular system^4^. Some small-scale randomized controlled trials (RCTs) have indicated that moderate consumption of dark chocolate can improve endothelial function^5^ and coronary artery vasodilation^6^, inhibit platelet adhesion^6^ and aggregation^7^, as well as reduce blood lipid levels^8^. However, the quality of these trials is reduced by bias introduced by differences in dosages of dark chocolate intake, lack of appropriate placebos, short follow-up periods and small sample sizes. In addition, regarding the impact of dark chocolate on blood pressure, some researchers have observed that dark chocolate intake can lower blood pressure in hypertensive patients as well as in healthy people^9,10^, while two early trials did not find any blood pressure-lowering effect of dark chocolate^11,12^. Moreover, these studies only revealed the association between dark chocolate intake and individual risk factors as well as certain aspects of the pathogenesis of CVDs, which do not necessarily imply an effect of dark chocolate on the reduction of the risk of CVDs. Currently, no clinical study with CVDs as hard outcomes proves causality between dark chocolate intake and the risk of CVDs. Uncertainty surrounds their relationship.

**Figure 1:**
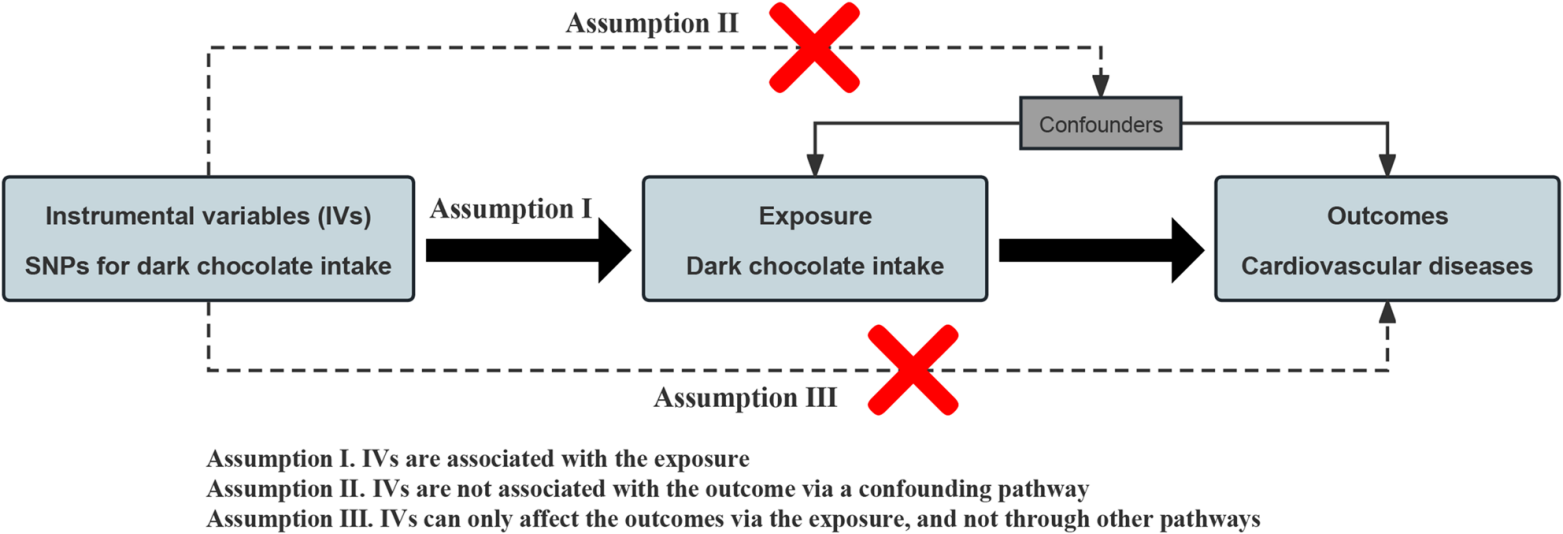
Illustration of the three instrumental variable assumptions for Mendelian randomization. *IVs* instrumental variables, *SNPs* single nucleotide polymorphisms.

In Mendelian randomization (MR) studies, genetic variation is used as a proxy for exposure factor, and MR have been widely used in recent years to explore the causality between exposure factors and diseases^13^. Because genetic variation is randomly inherited from parents to their children, it is unlikely to be related to potential confounders affecting the exposure-outcome association, nor is it subject to reverse causality^14^. However, MR studies also have limitations, as they must satisfy three instrumental variable (IV) assumptions (Fig. 1), otherwise bias may occur^15^.

In the past, MR studies have supported causality between certain foods, such as cheese and coffee, and partial CVDs^16,17^, but no studies have explored the causality between dark chocolate intake and any diseases. This two-sample MR study comprehensively investigated causality between genetically predicted dark chocolate intake and the risk of 12 CVDs.

## Methods

**Figure 2:**
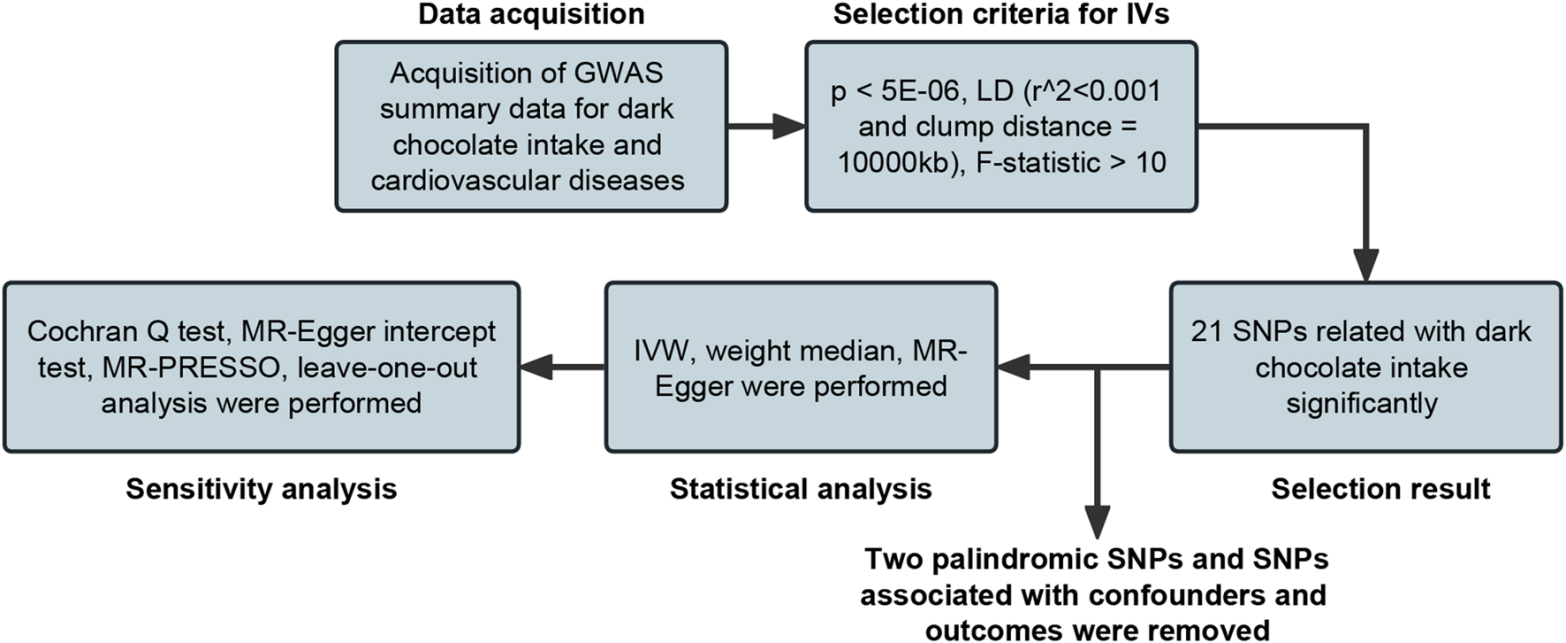
The main workflow of Mendelian randomization study revealing causality from dark chocolate intake on cardiovascular diseases. *IVs* instrumental variables, *LD* linkage disequilibrium, *SNPs* single nucleotide polymorphisms, *IVW* inverse variance weighted, *MR* Mendelian randomization.

The summarisation of the study design is shown in Fig. 2.

### Genetic instrumental variables associated with dark chocolate intake

The genome-wide association study (GWAS) summary-level data for dark chocolate intake were derived from a website (https://gwas.mrcieu.ac.uk/datasets) and analyzed by the MRC-IEU UK Biobank GWAS pipeline to perform GWAS on the UK Biobank genetic dataset. All resulting data have been quality controlled for the appropriate samples and single nucleotide polymorphisms (SNPs)^18^. The summary-level data of dark chocolate intake involved 64,945 European ancestry participants, and it’s GWAS ID is ukb-b-16139. The effect size of this GWAS data is reported in one-standard-deviation change.

We identified 21 independent SNPs as genetic instrumental variables (IVs) for predicting dark chocolate intake by setting the significance level of the association (p < 5 × 10^−6^) between SNPs and dark chocolate intake, as well as linkage disequilibrium (r^2^ < 0.001 and clump distance = 10,000 kb) (Table 1). In order to obtain a substantial number of SNPs and avoid excluding SNPs that might be genuinely associated with exposure, we did not employ the stringent traditional genome-wide significance threshold (p < 5 × 10^−8^)^19^. The association strength of the IVs was measured by computing the F-statistic as follows: $F=\frac{{R}^{2}\left(N-k-1\right)}{k\left(1-{R}^{2}\right)}$, where R^2^ is the variability in dark chocolate intake explained by each SNP, k is the number of SNPs and N is the GWAS sample size^20^. To calculate R^2^ for the extended 10 SNPs, we used the following formula:

**Table 1:** Summary information on SNPs associated with dark chocolate intake in the Mendelian randomization analyses.

SNP	Nearby gene	EA	OA	EAF	Beta	SE	p value	F-statistic
rs10799451	PRSS38	C	T	0.9529	-0.0308	0.0055	2.60E-08	31.0
rs61788541	DPYD	T	C	0.1761	-0.0139	0.0030	4.10E-06	21.2
rs75633229	NCKAP5	G	T	0.0076	0.0651	0.0135	1.30E-06	23.4
rs115832742	SORCS2	C	T	0.0090	0.0654	0.0123	9.90E-08	28.4
rs10462943	MAT2B	T	C	0.3667	-0.0119	0.0024	6.10E-07	24.9
rs114103245	SNCAIP	G	A	0.0099	0.0534	0.0116	4.30E-06	21.1
rs10806742	PARK2	C	T	0.3717	0.0112	0.0024	3.40E-06	21.6
rs145537395	SRSF3	A	G	0.0136	0.0491	0.0102	1.30E-06	23.4
rs259386	ADGB	C	T	0.6204	-0.0109	0.0024	4.70E-06	21.0
rs6916329	SLC2A12	C	T	0.0160	0.0455	0.0091	7.10E-07	24.6
rs78920357	RPS6KA2	C	T	0.0617	-0.0241	0.0050	1.80E-06	22.8
rs10273549	LSMEM1	A	G	0.0638	0.0224	0.0047	1.70E-06	22.9
rs6951020	RP11-507K12.1	C	T	0.9228	-0.0210	0.0043	1.10E-06	23.8
rs7008560	NCALD	A	T	0.0230	0.0376	0.0077	9.80E-07	24.0
rs17143632	GATA3	A	G	0.0059	0.0737	0.0149	8.00E-07	24.4
rs76124460	KCNMA1	A	G	0.0098	0.0612	0.0121	3.90E-07	25.7
rs71469535	ACER3	T	C	0.0159	0.0481	0.0100	1.40E-06	23.3
rs146635941	KLHL1	T	C	0.0189	0.0485	0.0093	2.10E-07	27.0
rs150993006	PCDH9	T	A	0.0108	0.0555	0.0119	3.10E-06	21.7
rs112868306	SERPINB8	C	T	0.0089	0.0573	0.0124	3.90E-06	21.3
rs5760827	KIAA1671	G	A	0.7775	-0.0135	0.0029	3.10E-06	21.7

*SNP* single-nucleotide polymorphism, *EA* effect allele, *OA* other allele, *EAF* effect allele frequency, *SE* standard error.

$${{\text{R}}}^{2}=\frac{2\times {\text{EAF}}\times \left(1-{\text{EAF}}\right)\times {\upbeta }^{2}}{2\times {\text{EAF}}\times \left(1-{\text{EAF}}\right)\times {\upbeta }^{2}+2\times {\text{EAF}}\times \left(1-{\text{EAF}}\right)\times {\text{N}}\times {{\text{SE}}}^{2}},$$

where EAF is the effect allele frequency, β is the estimated genetic effect of dark chocolate intake and SE is the standard error of the genetic effect^21^. The overall F-statistic (23.9) and the F-statistic for each SNP (Table 1) are both greater than the empirical strength threshold of 10^22^.

In addition, to ascertain whether these SNPs are associated with CVDs through other phenotypes (i.e., confounders), we also checked all SNPs in PhenoScanner (http://www.phenoscanner.medschl.cam.ac.uk/). The SNP rs75633229 is associated with systemic sclerosis, and several large cohort studies suggest that systemic sclerosis is a risk factor for multiple CVDs^23–25^. The SNP rs10806742 is associated with HbA1c^26^, but there is literature reporting that dark chocolate intake can significantly reduce HbA1c in diabetic children^27^, which may be a mediator of the beneficial effects of dark chocolate intake on CVDs. Therefore, we did not exclude it as an invalid IV. The SNP rs112868306 is related to glucocorticoid treatment, and several studies have reported that the use of glucocorticoids increases the risk of deep venous thrombosis (DVT), venous thromboembolism (VTE), and hypertension^28–30^. The SNP rs145537395 is directly associated with cerebrovascular disease. All SNPs for which the association with confounders and CVDs reaches the genome-wide significance threshold we set (p < 5 × 10^−6^) will be excluded before the MR analysis of corresponding outcome to ensure the validity of assumption II and III.

### GWAS summary-level data for CVDs

Summary-level data of coronary heart disease (CHD) were extracted from a large meta-analysis, which included nine contributing studies: deCODE, EPIC-CVD, German MI Family Study, Greek Coronary Disease cohort, HUNT, Mass General Brigham Biobank, TIMI, UK Biobank, and CARDIoGRAMplusC4D^31^. This meta-analysis comprised 181,522 cases and 984,168 controls. The summary-level data of atrial fibrillation (AF) were mainly from six contributing studies, including 60,620 cases and 970,216 controls of European ancestry^32^. Heart failure (HF) GWAS summary-level data came from 26 studies of the Heart Failure Molecular Epidemiology for Therapeutic Targets (HERMES), involving 47,309 cases and 930,014 controls of European ancestry^33^. The summary-level data of stroke was obtained from 29 studies of the MEGASTROKE consortium, including 40,585 cases and 406,111 controls of European ancestry^34^. Among them, 34,217 cases were defined as ischemic stroke, with the major etiological subtypes including large-artery atherosclerotic stroke, cardioembolic stroke, and small-vessel stroke.

We obtained summary-level data for essential hypertension (EH) (92,462 cases/265,626 controls), non-rheumatic valve disease (20,772 cases/286,109 controls), non-ischemic cardiomyopathy (9926 cases/303,607 controls), transient ischemic attack (TIA) (18,398 cases/342,294 controls), VTE (19,372 cases/357,905 controls), myocardial infarction (MI) (24,185 cases/313,400 controls) and DVT of lower extremities (9109 cases/324,121 controls) from the FinnGen consortium R9 release^35^, which we downloaded through Google Cloud Storage. The FinnGen consortium identifies cases of these diseases primarily using the International Classification of Diseases codes from the 8th, 9th, and 10th revisions.

There is some degree of sample overlap in the GWAS summary-level data for exposure and CHD. Statistical analysis for all outcome GWAS data was conducted using logistic regression model, and the effect sizes were computed in unit of log odds. Information on all outcome summary-level data used in this study is provided in Supplementary Tables 1, 2.

### Statistical analysis

We harmonized the datasets of dark chocolate intake and CVDs to align the direction of alleles of the SNPs for both, while removing palindromic and incompatible SNPs.

The Wald ratio method estimates how exposure affects the outcome by calculating the ratio of the outcome effect and the exposure effect for each SNP, while the inverse-variance weighted (IVW) method is a meta-analysis of the Wald ratios for each SNP^36,37^. Based on the magnitude of heterogeneity, the IVW method using different models was used as the main method for MR analysis. The IVW method provides the most efficient estimate of causality, but assumes that all IVs are valid, and therefore is affected by the actual pleiotropy^37,38^. Therefore, we also employed MR-Egger method and weighted median method as auxiliary analytical approaches. The MR-Egger method corrects for pleiotropy and produces effect estimates that are not biased by violations of the IV assumption^39^. The weighted median method requires at least 50% of the weight to come from valid IVs in order to yield consistent estimates of causal effect^40^. Due to the involvement of multiple outcomes in our study, we employ Bonferroni correction to account for multiple comparisons. We deemed a p-value of less than 0.004 (0.05/12 outcomes) as statistically significant. Any p-value between 0.004 and 0.05 indicates a suggestive association. Finally, we will perform statistical power calculations using an online tool that is available at https://shiny.cnsgenomics.com/mRnd/^41^.

### Sensitivity analysis

To assess the robustness of the results of the MR analysis, we performed sensitivity analyses using several methods. First, Cochran's Q test was employed to identify heterogeneity among the effect estimates of each SNP. If the p-value exceeds 0.05, indicating the absence of heterogeneity, the IVW method will utilize the fixed-effects model. Otherwise, the random-effects model will be used. Second, MR-Egger intercept test was used to examine horizontal pleiotropy, which is crucial. If the test indicates the presence of pleiotropy (p < 0.05), then the results from the IVW method will be unreliable^39^. Third, MR-PRESSO is capable of identifying SNPs with horizontal pleiotropy (i.e., outliers), and can assess whether there are significant variations in causal effect estimates before and after outliers removal^42,43^. The results of the MR-Egger intercept test and MR-PRESSO can further validate assumption II and III. Fourth, leave-one-out analysis was performed to assess the impact of individual SNPs on the MR estimates. The number of SNPs selected in our study is not large, and the leave-one-out analysis has a good performance.

The significant causality is defined as follows: (1) the P-value of the IVW method must be less than 0.004; (2) the estimates of the three MR analysis methods must have consistent directions; (3) the MR-Egger intercept test should not show horizontal pleiotropy (P > 0.05); (4) the MR-PRESSO global test should not be significant (P > 0.05). In this study, we utilized the "TwoSampleMR" and "MRPRESSO" packages in R software version 4.2.2 (https://www.r-project.org/) for all statistical analyses. All p-values in this study are two-sided.

## Results

**Figure 3:**
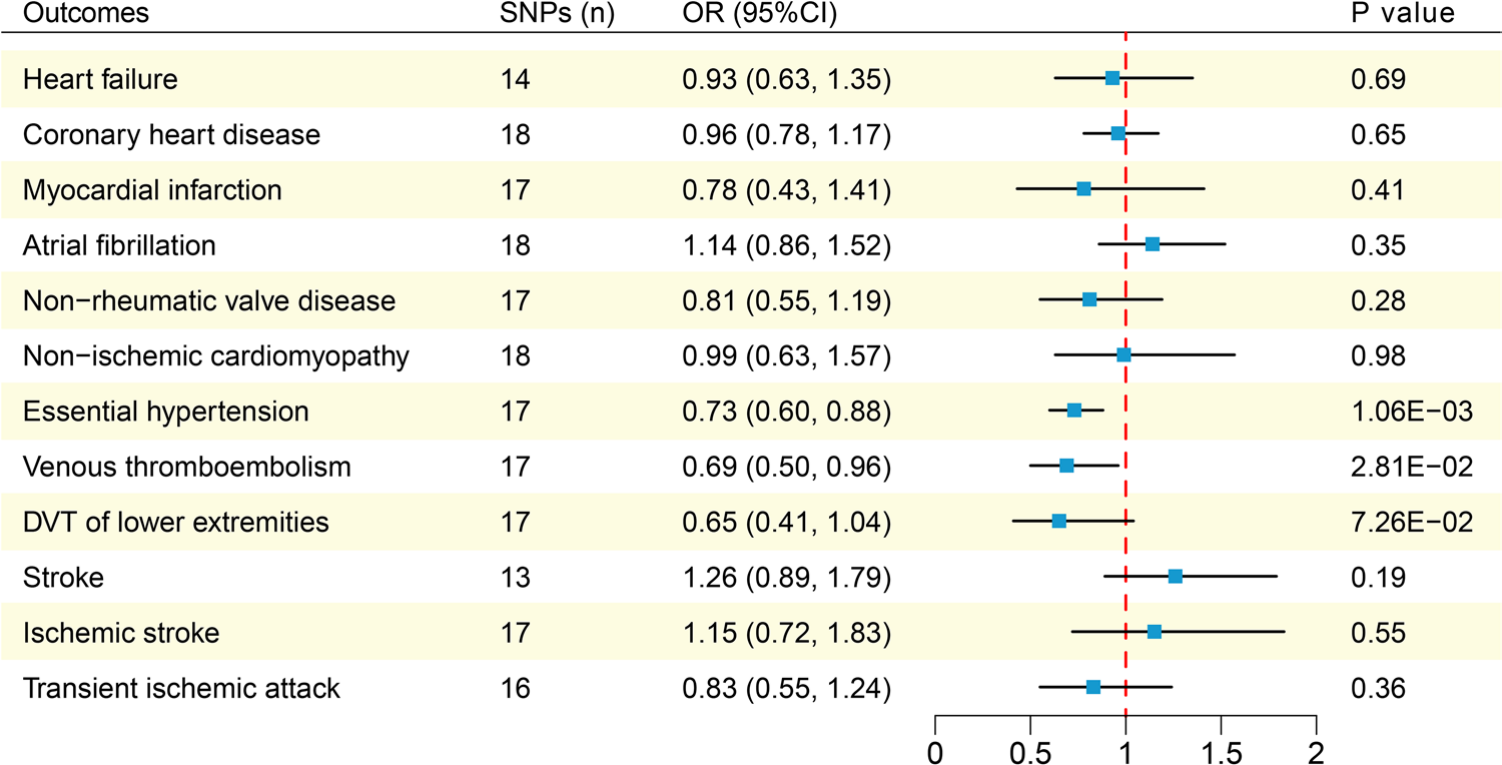
Mendelian randomization analysis estimates of dark chocolate intake and the risk of cardiovascular diseases. *DVT* deep venous thrombosis, *SNPs* single nucleotide polymorphisms, *OR* odds ratio, *CI* confidence interval.

The SNPs not included in the MR analysis for each outcome are shown in Supplementary Table 3. The causality between dark chocolate intake and the risk of CVDs estimated by MR analysis is shown in Fig. 3. The IVW analysis results showed that the dark chocolate intake per standard deviation increase was significantly inversely associated with the risk of EH (odds ratio (OR) = 0.73; 95% confidence interval (CI), 0.60–0.88; p = 1.06 × 10^−3^), and there was a suggestive negative association with VTE (OR = 0.69; 95% CI, 0.50–0.96; p = 2.81 × 10^−2^). In contrast, there was no association observed between dark chocolate intake and HF, CHD, MI, AF, non-rheumatic valve disease, non-ischemic cardiomyopathy, DVT of lower extremities, stroke, ischemic stroke, as well as TIA. The results of the weighted median and MR-Egger methods are shown in Table 2. The MR-Egger intercept test showed evidence of horizontal pleiotropy in the MR analysis result for VTE (intercept = 1.92 × 10^−2^; p = 0.02), and the Cochran's Q test revealed heterogeneity in the result for MI (p = 8.66 × 10^−4^) (Table 3). In the MR-PRESSO analysis, only the result for MI exhibited outliers, and the MR-PRESSO global test indicated the presence of horizontal pleiotropy (p = 7 × 10^−4^) (Table 3). The statistical power of the OR for the association between dark chocolate intake and EH has reached 100%, whereas the statistical power of the ORs for the association with other outcomes is all below 80%.

**Table 2:** MR-Egger and weighted median methods for the associations of dark chocolate intake with cardiovascular diseases.

Outcomes	MR-Egger		Weighted median	
	OR (95% CI)	pval	OR (95% CI)	pval
Heart failure	1.20 (0.48, 3.00)	0.71	1.07 (0.62, 1.86)	0.80
Coronary heart disease	0.99 (0.69, 1.42)	0.96	0.96 (0.72, 1.28)	0.78
Myocardial infarction	0.75 (0.28, 2.03)	0.58	1.04 (0.57, 1.92)	0.89
Atrial fibrillation	1.28 (0.68, 2.40)	0.45	1.00 (0.65, 1.54)	0.99
Non-rheumatic valve disease	0.54 (0.29, 1.01)	0.07	0.77 (0.44, 1.33)	0.35
Non-ischemic cardiomyopathy	1.01 (0.49, 2.06)	0.98	1.27 (0.64, 2.51)	0.49
Essential hypertension	0.65 (0.48, 0.87)	0.01	0.70 (0.54, 0.90)	6.26E-03
Venous thromboembolism	0.41 (0.24, 0.69)	4.53E-03	0.68 (0.42, 1.10)	0.12
DVT of lower extremities	0.36 (0.17, 0.74)	0.01	0.55 (0.28, 1.09)	0.09
Stroke	1.01 (0.53, 1.91)	0.98	1.07 (0.66, 1.74)	0.78
Ischemic stroke	0.91 (0.34, 2.39)	0.84	1.08 (0.56, 2.08)	0.82
Transient ischemic attack	0.51 (0.24, 1.08)	0.10	0.71 (0.40, 1.28)	0.25

*DVT* deep venous thrombosis, *OR* odds ratio, *CI* confidence interval.

**Table 3:** Three sensitivity analyses for the associations of dark chocolate intake with cardiovascular diseases.

Outcomes	Q pval	Intercept (pval)	Pval of MR-PRESSO global test
Heart failure	0.11	-5.59E-03 (0.54)	0.09
Coronary heart disease	0.41	-9.83E-04 (0.81)	0.50
Myocardial infarction	8.66E-04	1.06E-03 (0.93)	7E-04
Atrial fibrillation	0.08	-2.80E-03 (0.68)	0.10
Non-rheumatic valve disease	0.71	1.27E-02 (0.12)	0.56
Non-ischemic cardiomyopathy	0.81	-4.77E-04 (0.96)	0.85
Essential hypertension	0.87	4.26E-03 (0.33)	0.72
Venous thromboembolism	0.12	1.92E-02 (0.02)	0.18
DVT of lower extremities	0.55	2.20E-02 (0.052)	0.44
Stroke	0.35	5.64E-03 (0.41)	0.40
Ischemic stroke	0.24	5.50E-03 (0.57)	0.30
Transient ischemic attack	0.10	1.51E-02 (0.13)	0.15

*Q_pval* p value corresponding to Cochran’s Q test, *Intercept* the intercept of MR-Egger regression.

The scatter plot and forest plot of individual SNPs showing the association between dark chocolate intake and CVDs are presented in Supplementary Fig. 1 and Supplementary Fig. 2, respectively, providing a more visual representation of the results in another form. Most of the funnel plots for the results are relatively symmetrical and did not indicate significant evidence of heterogeneity or pleiotropy (Supplementary Fig. 3). The results of leave-one-out analysis are shown in Supplementary Fig. 4, indicating that most estimates were not greatly influenced by any single SNP.

## Discussion

In this two-sample MR study, based on our defined criteria for significant causality, we found that genetically predicted dark chocolate intake was associated with a lower risk of EH, but not with HF, CHD, MI, AF, non-rheumatic valvular heart disease, non-ischemic cardiomyopathy, DVT of the lower extremities, stroke, ischemic stroke, and TIA. Limited evidence supports a suggestive negative association between dark chocolate intake and the risk of VTE, and the analysis results show horizontal pleiotropy, making it unable to establish a causality between them.

Dark chocolate is rich in substances such as flavanols, methylxanthines, and caffeine, with flavanols being the main reason for its cardiovascular benefits. Studies have shown that flavanols can activate the NO synthase in endothelial cells, leading to the release of NO, which then activates the guanylate cyclase in smooth muscle cells, increasing the levels of cyclic guanosine monophosphate, and subsequently causing a decrease in intracellular calcium ion concentration, resulting in vasodilation^44^. A RCT involving 45 participants showed that acute ingestion of dark chocolate compared to placebo can improve endothelial function (measured as flow-mediated dilation)^45^. In another RCT involving 22 heart transplant recipients, intake of dark chocolate significantly increased coronary artery diameter and endothelium-dependent coronary vasodilation^6^. Previous studies have shown that flavanols has physiological effects similar to aspirin in inhibiting cyclooxygenase^46^. Additionally, flavanols can inhibit platelet aggregation by promoting the release of NO^44^. Two small-sample RCTs indicate that dark chocolate can significantly reduce shear stress-dependent platelet adhesion and platelet aggregation^6,7^. In addition, flavanols can regulate the production of pro-inflammatory cytokines. The NO released by flavanols can also inhibit the recruitment of white blood cells and the aggregation of platelets to the site of inflammation, thereby exerting local anti-inflammatory activity^47^, which is particularly important for delaying the development of atherosclerosis. Previous studies have found that inflammation is an important risk factor for CVDs, especially atherosclerosis-related diseases^48,49^. Regarding the lipid-lowering effects of dark chocolate, meta-analyses have shown that consumption of dark chocolate and cocoa products can lower levels of low-density lipoprotein cholesterol and total cholesterol, while increasing high-density lipoprotein cholesterol levels^8,50^. However, in this MR study, we did not find any evidence for the reduction of the risk of 11 outcomes excluding EH, which is not contradictory to previous findings. Based on the evidence from intervention studies mentioned above, dark chocolate indeed provides some cardiovascular benefits. However, the pathophysiological mechanisms underlying the development and progression of CVDs are highly complex, and these studies only revealed individual risk factors and certain aspects of the pathogenesis of CVDs affected by dark chocolate intake. It cannot be concluded that dark chocolate intake reduces the risk of CVDs. The cardiovascular benefits of dark chocolate intake may not be sufficient to reduce the risk of these diseases. Moreover, most of these small-sample controlled trials have some limitations, which lead to a low level of reliability of their conclusions.

The research results on the relationship between dark chocolate and blood pressure were controversial 10 years ago^51^. However, the latest meta-analysis involving 31 studies showed that consuming cocoa beverages or chocolate for more than 2 weeks was associated with a reduction in systolic and diastolic blood pressure. Nevertheless, the overall effect size of blood pressure reduction was too small to be considered clinically significant^52^. A cross-sectional study involving 14,310 Jordanian adults also showed that dark chocolate intake has a significant beneficial effect on the blood pressure of healthy adults^53^. The mechanism of blood pressure reduction by dark chocolate may be related to the increase of NO^44^, and there is also evidence that flavanols have an inhibitory effect on angiotensin-converting enzyme activity in vitro^54^. In this study, we have established a clear association between dark chocolate intake and a reduced risk of EH, which aligns to some extent with the historical overall tendency that suggests dark chocolate reduces blood pressure^52,53^. However, it is important to note that lowering blood pressure does not completely equate to reducing the risk of EH, as many risk factors for hypertension may lead to physiological and histological changes in the body that cannot be reversed by simply lowering blood pressure.

In this MR study, we used data on EH from the FinnGen consortium, rather than data on hypertension in general. As the sample of hypertension data includes cases of secondary hypertension, which is essentially the manifestation of kidney disease, renal vascular disease, and some endocrine diseases (such as pheochromocytoma, Cushing's syndrome, and aldosteronism) in the cardiovascular system, our study does not investigate the causality between dark chocolate intake and these diseases. In addition, some methodological researchers of MR oppose interpreting causal effect estimates as expected effects of intervening on the exposure factor in a clinical setting, and even recommend against making causal effect estimates^55^. Therefore, we do not recommend that everyone prevent EH by consuming dark chocolate, but rather suggest that individuals at risk for EH can replace their usual unhealthy snacks with dark chocolate. The risk factors for EH include a family history of EH, lack of physical activity, obesity, smoking, alcohol consumption, high-salt and high-fat diet, and advanced age^56^. By observing the presence of these risk factors, individuals at risk for EH can be identified.

This study has several significant strengths. First, there have been no MR studies or clinical studies analyzing the causality between dark chocolate intake and the risk of CVDs in the past. Second, this MR analysis utilized the latest exposure and outcome GWAS summary-level data, comprehensively analyzed the causality between dark chocolate intake and multiple CVDs, maximally avoided the influence of confounders and reverse causality, and used multiple sensitivity analysis methods to verify the causality. Third, MR studies reveal the total effect, which in this study refers to the overall impact of all components (including potentially harmful sugar and fat for health) in dark chocolate on CVDs. However, many controlled trials use placebos with similar components to dark chocolate, some of which only differ in their flavanols content^52^, which to some extent reduces the credibility of previous research conclusions.

This study also has several limitations. First, the sample size of the exposure data is not large enough, and the screened SNPs did not reach the traditional genome-wide significance threshold (P < 5 × 10^−8^). But all of the IVs have an F-statistic greater than 10, indicating that these SNPs can be considered as effective IVs. The results of sensitivity analysis also demonstrate the robustness of the estimated causal effects for the vast majority of outcomes. Second, a considerable number of SNPs were lost in the summary-level data for HF and stroke, which may have some impact on the analysis results. Third, the sample populations in the summary-level data for the exposure and outcome are of European ancestry, which makes it difficult to generalize the study results to other populations. Fourth, the summary-level data we used did not stratify for certain factors, which only allowed for general analysis and could not investigate the relationship between the risk of CVDs and age, gender of the population, as well as the amount of dark chocolate intake. Fifth, there is some degree of overlap between the GWAS sample for CHD and the exposure. While one study indicates that when the sample size is large, it is safe to conduct one-sample MR using two-sample MR methods^57^, potential biases arising from sample overlap should not be overlooked. Sixth, the GWAS sample of CHD cover 5% of the non-European population, and the existence of population stratification may confound the association between genetic variants and outcome. Due to the last two limitations, the results about CHD may be unreliable.

## Conclusion

This two-sample MR study revealed causality between dark chocolate intake and reduced risk of EH. However, the causality with VTE could not be established due to insufficient evidence, and there is no causality observed with other CVDs. Our findings have some implications for the prevention of EH in the population. However, further clinical research is still needed to explore the causality between dark chocolate intake and the risk of CVDs.

### Supplementary Information

Supplementary Information.

## Data Availability

GWAS summary-level data from all database sources in this study are available from FinnGen Consortium (https://r9.finngen.fi/) and IEU OpenGWAS project (https://gwas.mrcieu.ac.uk/datasets). GWAS summary-level data for CHD, AF, HF, stroke and ischemic stroke can be obtained from four large-scale meta-analysis articles^31–34^. We clarified that the human data used in this study are available from publicly available databases and large meta-analysis articles. We have refined the data availability statement and clarified that the human data used in our studies are publicly available.
