# Supplementary material for: Dark chocolate intake and cardiovascular diseases: a Mendelian randomization study
Source: Sci Rep. 2024 Jan 10;14:968. doi: 10.1038/s41598-023-50351-6 (PMC10781976; doi:10.1038/s41598-023-50351-6)
Supplement: Supplementary file 1 — Supplementary Information. [file 41598_2023_50351_MOESM1_ESM.pdf]

Supplemental materials

Dark Chocolate Intake and Cardiovascular Diseases: A Mendelian Randomization Study

Author names: Juntao Yang, Jiedong Zhou, Jie Yang, Haifei Lou, Bingjie Zhao, Jufang Chi, Weiliang Tang

Corresponding author: Weiliang Tang. Department of Cardiology, Shaoxing People's Hospital, Shaoxing, Zhejiang 312000, P. R. China; E-mail: twl-sxyz@163.com

Supplementary Table 1. Information of outcome data from the FinnGen consortium used in this study.

Outcome	Data code	Year	Ethnicity	Cases	Controls	Number of SNPs	Disease definition		
							ICD-10	ICD-9	ICD-8
DVT of lower extremities	I9_PHLETHROMBDVTLOW	2023	Finnish	9,109	324,121	20,169,198	I80.3#, I80.20#, I80.29#	4511A/4510A/4512A	4510
Essential hypertension	I9_HYPTENSESS	2023	Finnish	92,462	265,626	20,169,845	I10	4039A/4019X	401/402/403/404
Non-rheumatic valve diseases	I9_NONRHEVALV	2023	Finnish	20,772	286,109	20,168,219	I34, I35, I36, I37	424	39[4-6]/424, *
Non-ischemic cardiomyopathy	I9_NONISCHCARDMYOP	2023	Finnish	9,926	303,607	20,168,549	-	-	-
Transient ischemic attack	I9_TIA	2023	Finnish	18,398	342,294	20,169,914	G45, §	435	435
Venous thromboembolism	I9_VTE	2023	Finnish	19,372	357,905	20,170,236	I26, I80, O87.1, O88.2, †	415/451/6713/6714/ 6732, ‡	450/451/671/6739
Myocardial infarction	I9_MI_STRICT	2023	Finnish	24,185	313,400	20,169,327	I21, I22, £	410	410

DVT, deep venous thrombosis; SNPs, single nucleotide polymorphisms; ICD, International Classification of Disease.
*Exclude 4249, 3940, 3950, 3960 and 3970. §Exclude G45.4. †Exclude I80.0#. ‡Exclude 4510. £Exclude I21.90, I21.97, I22.90, I22.97.

Supplementary Table 2. Information of other outcome data used in this study.

Outcomes	Year	Ethnicity	Consortium	Cases	Controls	Number of SNPs
Coronary heart disease	2022	Over 95% of European	-	181,522	1,165,690	20,073,070
Atrial fibrillation	2018	European	AFGen	60,620	970,216	33,519,037
Heart failure	2020	European	HERMES	47,309	930,014	7,773,021
Stroke	2018	European	MEGASTROKE	40,585	406,111	7,633,440
Ischemic stroke	2018	European	MEGASTROKE	34,217	406,111	8,296,492

SNPs, single nucleotide polymorphisms.

Supplementary Table 3. The SNPs not included in the MR analysis for each outcome.

Outcome	SNPs lost in outcome data	SNPs associated with confounders and outcomes	Palindromic SNPs
Coronary heart disease	-	rs75633229	rs150993006, rs7008560
Atrial fibrillation	-	rs75633229	
Heart failure	rs115832742, rs17143632, rs150993006, rs146635941, rs112868306	rs75633229	
Stroke	rs115832742, rs71469535, rs150993006, rs146635941, rs112868306	rs75633229, rs145537395	
Ischemic stroke	rs150993006	rs75633229, rs145537395	
Myocardial infarction	rs5760827	rs75633229	
DVT of lower extremities	rs5760827	rs112868306	
Essential hypertension	rs5760827	rs112868306	
Non-rheumatic valve disease	rs5760827	rs75633229	
Non-ischemic cardiomyopathy	rs5760827	-	
Transient ischemic attack	rs5760827	rs145537395, rs75633229	
Venous thromboembolism	rs5760827	rs112868306	

SNPs, single-nucleotide polymorphisms; DVT, deep venous thrombosis.

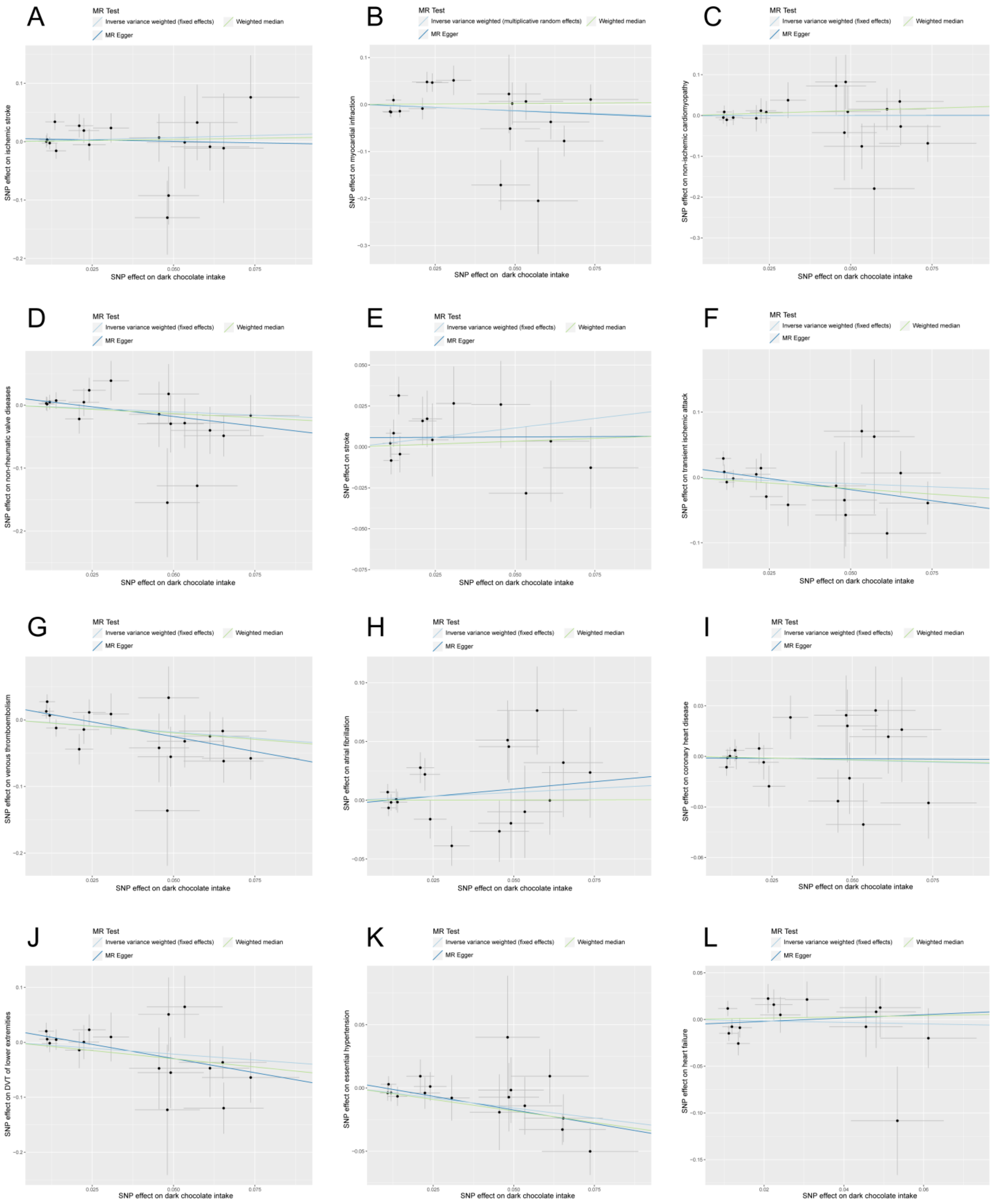

Supplementary Figure 1. Scatter plot for the association of dark chocolate intake with (A) ischemic stroke, (B) myocardial infarction, (C) non-ischemic cardiomyopathy, (D) non-rheumatic valve disease, (E) stroke, (F) transient ischemic attack, (G) venous thromboembolism, (H) atrial fibrillation, (I) coronary heart disease, (J) DVT of lower extremities, (K) essential hypertension and (L) heart failure.

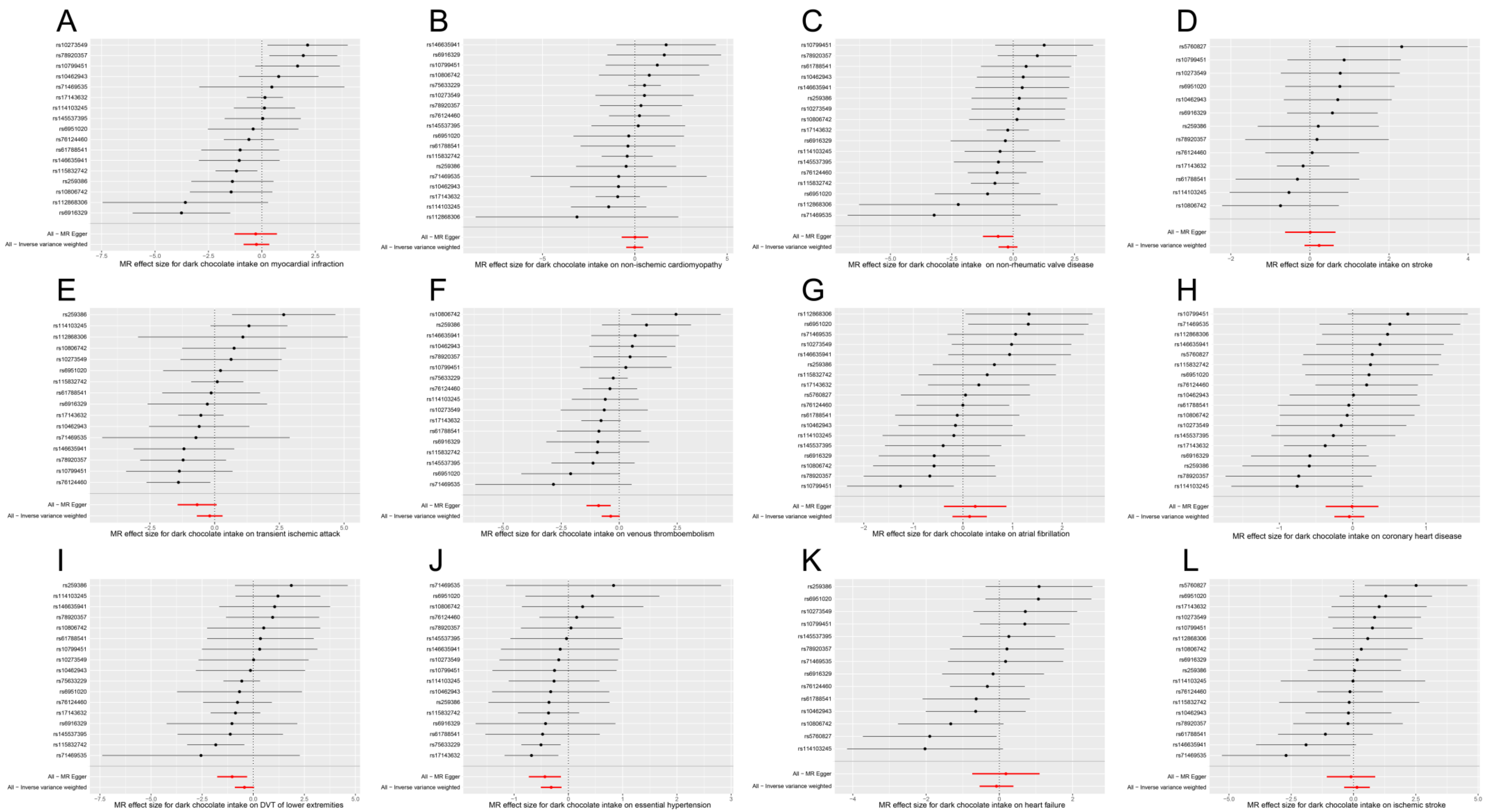

Supplementary Figure 2. Forest plot of individual SNPs for the association of dark chocolate intake with (A) myocardial infarction, (B) non-ischemic cardiomyopathy, (C)non-rheumatic valve disease, (D) stroke, (E) transient ischemic attack, (F) venous thromboembolism, (G) atrial fibrillation, (H) coronary heart disease, (I) DVT of lower extremities, (J) essential hypertension, (K) heart failure and (L) ischemic stroke.

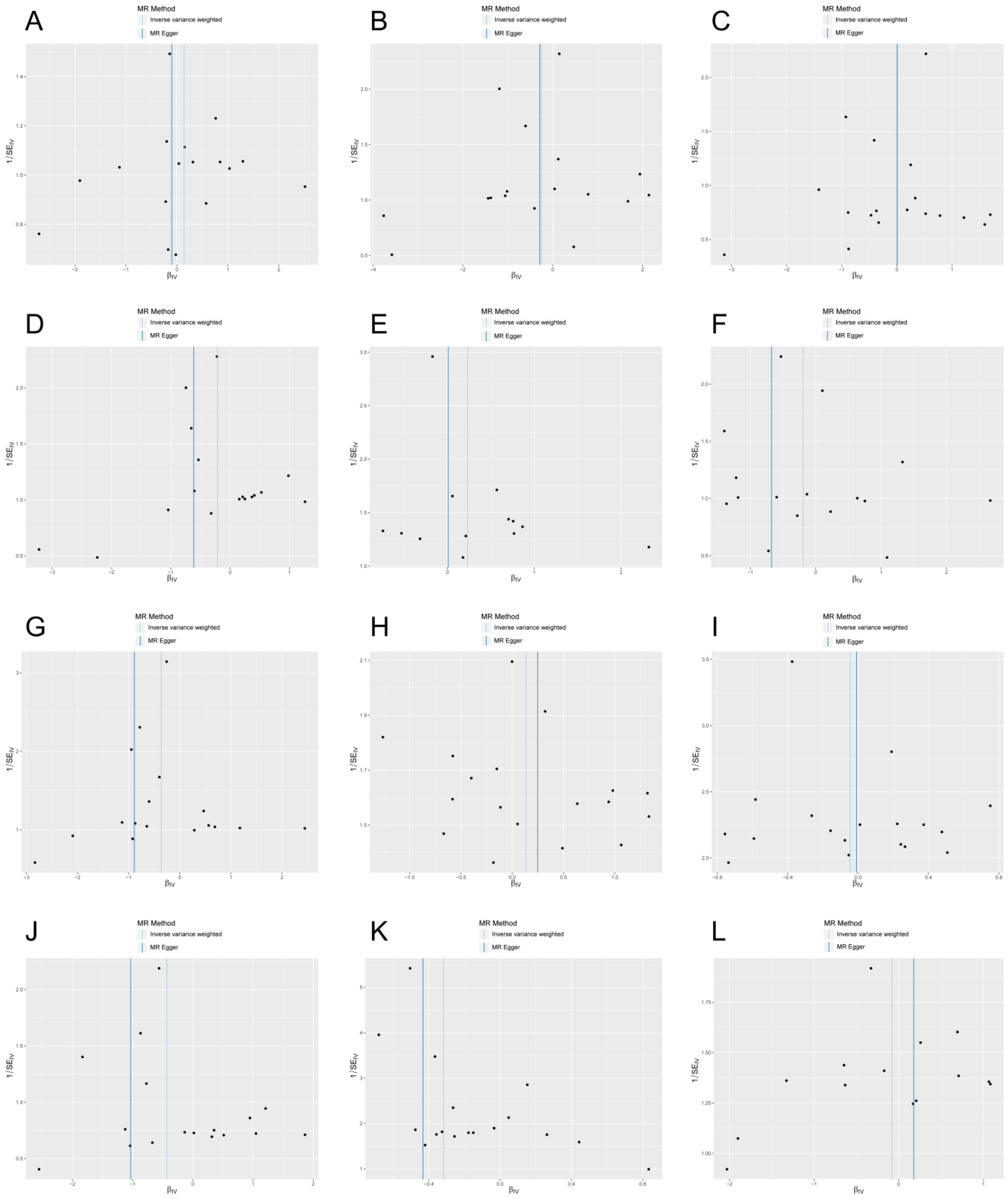

Supplementary Figure 3. Funnel plot for the association of dark chocolate intake with (A) ischemic stroke, (B) myocardial infarction, (C) non-ischemic cardiomyopathy, (D) non-rheumatic valve disease, (E) stroke, (F) transient ischemic attack, (G) venous thromboembolism, (H) atrial fibrillation, (I) coronary heart disease, (J) DVT of lower extremities, (K) essential hypertension and (L) heart failure.

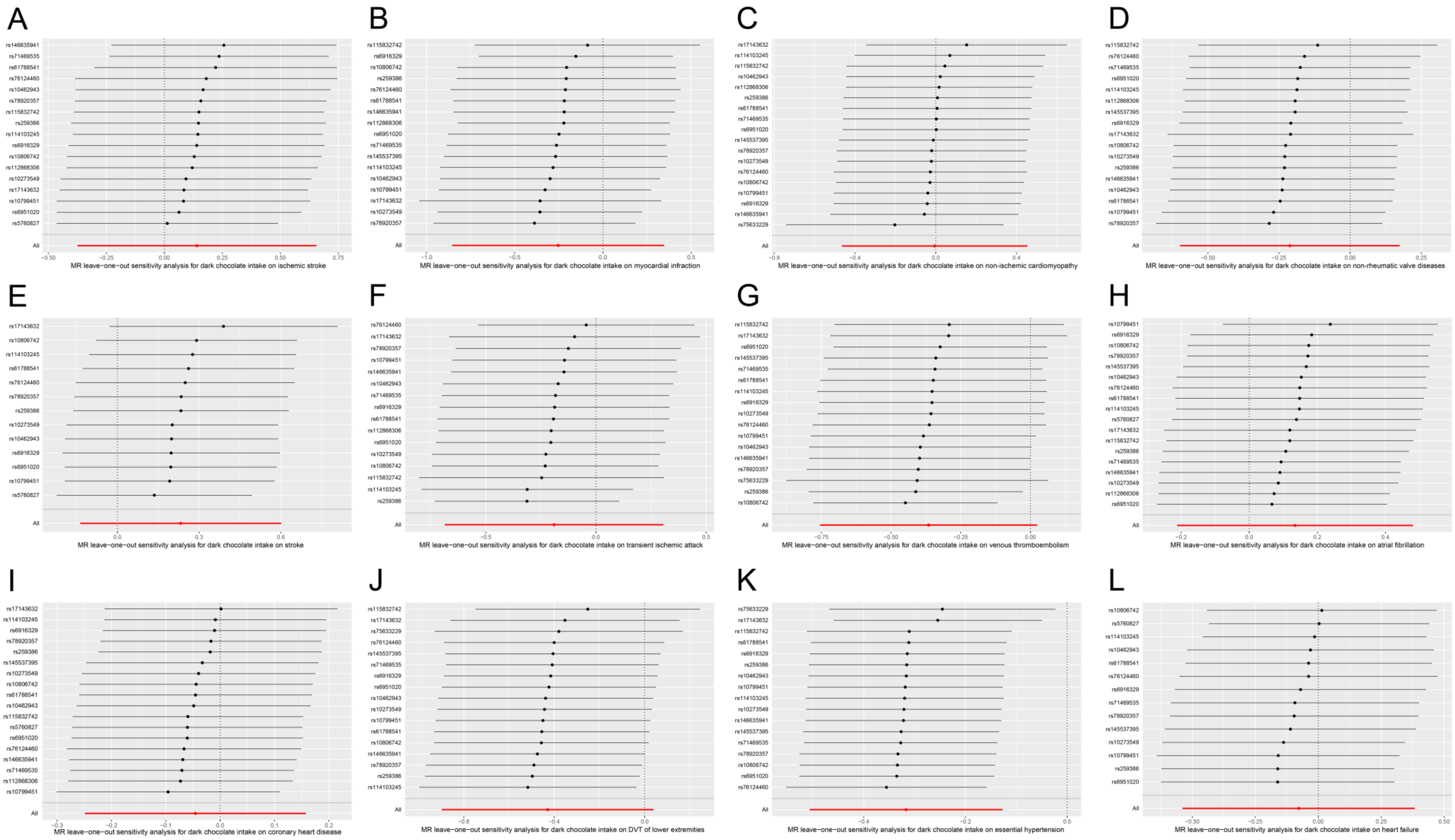

Supplementary Figure 4. Leave-one-out sensitivity analysis for the association of dark chocolate intake with (A) ischemic stroke, (B) myocardial infarction, (C) non-ischemic cardiomyopathy, (D) non-rheumatic valve disease, (E) stroke, (F) transient ischemic attack, (G) venous thromboembolism, (H) atrial fibrillation, (I) coronary heart disease, (J) DVT of lower extremities, (K) essential hypertension and (L) heart failure.
